# Genome-Wide Landscape of North-Eastern European Populations: A View from Lithuania

**DOI:** 10.3390/genes12111730

**Published:** 2021-10-28

**Authors:** Alina Urnikyte, Alma Molyte, Vaidutis Kučinskas

**Affiliations:** 1 Department of Human and Medical Genetics, Institute of Biomedical Sciences, Faculty of Medicine, Vilnius University, Santariškiu St. 2, LT-08661 Vilnius, Lithuania; alma.molyte@mf.vu.lt (A.M.); vaidutis.kucinskas@mf.vu.lt (V.K.); 2 Department of Information Systems, Faculty of Fundamentals Sciences, Vilnius Gediminas Technical University, Saulėtekio Al. 11, LT-10223 Vilnius, Lithuania

**Keywords:** population structure, effective population size, positive selection, Balto-Slavic populations

## Abstract

There are still several unanswered questions regarding about ancient events in the Lithuanian population. The Lithuanians, as the subject of this study, are of great interest as they represent a partially isolated population maintaining an ancient genetic composition and show genetic uniqueness in European comparisons. To elucidate the genetic relationships between the Lithuania and North-Eastern European and West Siberian populations, we analyzed the population structure, effective population size, and recent positive selection from genome-wide single nucleotide polymorphism (SNP) data. We identified the close genetic proximity of Lithuanians to neighboring populations (Latvians, Estonians, Belarusians) and in part with West and South Slavs (Poles, Slovaks, and Slovenians), however, with particular genetic distinctiveness. The estimated long-term *Ne* values ranged from ~5900 in the Estonian population to ~2400 in the South Russian population. The divergence times between the Lithuanian and study populations ranged from 240 to 12,871 YBP. We also found evidence of selection in 24 regions, 21 of which have not been discovered in previous analyses of selection. Undoubtedly, the genetic diversity analysis of geographically specific regions may provide new insights into microevolutionary processes affecting local human populations.

## 1. Introduction

Recent genetic research has revealed that Lithuanians represent a partially isolated population maintaining an ancient genetic composition and genetic uniqueness within the European context [1]. Lithuania is a country in northeastern Europe surrounded by the Baltic sea in the west; Latvia and Estonia in the North; Russia in the East; Belarus in the Southeast; and Poland in the South. Two main regions Aukstaitija (West, South, and East) and Zemaitija (North, West, and South) can currently be distinguished in the Lithuanian population. Lithuania is a complex amalgam between the former Baltic tribes speaking the most archaic Indo-European language [2]. However, not all Baltic people speak the same language, as Estonians belong to the Finno-Ugric branch of the Uralic languages as well as Finns. A main question has arisen about the impact of Slavs and Finno-Ugrian populations on Balts and their genetic relatedness. Language might not always be the most important factor revealing relatedness of geographically close neighbors. The early stages of colonization of the Baltic countries are not well known [3]. The first settlers of Lithuania arrived in West Lithuania along the Baltic Sea after the last glaciation around 11,000 years before present [4]. These people arrived from Western Europe and are related to hunter–gatherers (HG) [5]. The first Baltic Coastal culture in the territory of Lithuania was formed through the interaction of autochthonous populations and Indo-Europeans in the late Neolithic [6]. It has been suggested that from the middle Neolithic, about 6000–5000 years before present (YBP), the Finno-Ugric people arrived at the eastern coast of the Baltic region. The archaeological, linguistic, and genetic evidence shows uncertain influence of the Finno-Ugric on the Balts [4,7]. In accordance with Cesnys et al. (2004) [7], when considering LW*B gene and R408W phenylketonuria mutation together with VNTR and STR haplotypes, Balts are admixed with neighboring Slavic and Finno-Ugric populations. Additionally, Y chromosome STR haplotypes showed significant differences between the Lithuanian and Estonian populations, suggesting different origins or split time before Indo-Europeanization took place in the Eastern Baltic region [8]. Moreover, Laitinen et al.’s (2002) analysis of Y chromosome specific biallelic markers revealed similarities between both Lithuanians and Latvians and the Finno-Ugric Estonians and Mari [3]. Otherwise, mtDNA diversity analysis also highlighted similarities of Lithuanians not only with Finno-Ugrians, but also with the Slavs, Indo-Europeans of Northern and Eastern Europe [9]. Until the late Middle Ages, the Eastern Baltic region was one of the most isolated places in Europe [8]. Furthermore, when the Roman Empire fell in the 5th century, the Eastern Baltic region was bypassed by the population movements of the Migration Period [1,10], which permitted the most archaic of all the living speaking Indo-European languages to survive.

Our aim was to assess the genetic relationships between the Lithuanian and North-Eastern European and West Siberian populations by analyzing population structure, effective population size, and recent positive selection from whole-genome SNP data. To the best of our knowledge, little attention has been paid to the Lithuanian population elucidating genetic relationship between the Baltic people, who appeared as reference populations consisting of few individuals [11,12]. 

## 2. Materials and Methods

### 2.1. Samples

A dataset of 425 Lithuanian individuals who indicated at least three generations of Lithuanian nationality was used for analysis. The average age of the participants was ±53 years. In accordance with the Declaration of Helsinki, informed consent was obtained from all the study participants. Genomic DNA was obtained from whole blood using either a standard phenol-chloroform method of extraction or the automated DNA extraction platform TECAN Freedom EVO (TECAN Group Ltd., Männedorf, Switzerland). A NanoDropR ND-1000 spectrophotometer (NanoDrop Technologies Inc., Wilmington, DE, USA) was used to assess DNA concentration and quality. This work is part of the ANELGEMIA project, which was approved by the Vilnius Regional Research Ethics Committee No. 2020/6-1243-724, date: 22 June 2020. 

### 2.2. Genotyping

The Lithuanian samples were genotyped on an Illumina HumanOmniExpress-12v1.1 (296 samples) and Infinium OmniExpress-24 (129 samples) arrays (Illumina, San Diego, CA, USA), which include an overlap of 707,138 SNPs. Genotyping cells and quality control were performed according to the standard manufacturer’s recommendations. The data were filtered with PLINK (v1.07) to remove individuals and SNPs with >10% missing data and SNPs with minor allele frequency (MAF) <0.01. SNPs with deviations from Hardy–Weinberg equilibrium (*P* < 10^−4^) were also filtered out. We obtained 424 Lithuanian samples and 532,836 autosomal SNPs. To study genetic variation in broader geographical and historical context, we merged our genotyping data with the genome wide SNP data obtained from Kushniarevich et al. (2015) [13] and from Tambets et al. (2018) [12]. We generated a pooled dataset of 105,853 SNPs from a total of 596 individuals from Lithuania (*N* = 425), Latvia (*N* = 7), Estonia (*N* = 42), Belarus (*N* = 8), Central Russia (*N* = 2), South Russia (*N* = 4), Finland (*N* = 20), Khanty (*N* = 10), Mansi (*N* = 23), Poland (*N* = 5), Saami (*N* = 13), Saami Kola (*N* = 8), Slovakia (*N* = 15), and Slovenia (*N* = 15). 

### 2.3. Admixture and Principal Component Analysis

Principal component analysis (PCA) was performed with SmartPCA from EIGENSOFT (v7.2.1) [14] on the independently pruned SNPs. SNPs in linkage equilibrium were removed with the indep-pairwise (200 25 0.4) option of PLINK (v1.07) [15]. After LD pruning, we left with 78,249 SNPs. Genetic similarity between the Lithuanian genotyped individuals was inferred through the kinship coefficient, which was estimated with KING v.2.113 [16]. Individuals with 2nd degree relatives (kinship coefficient > 0.0084) and PCA outliers (in total 18 individuals) were removed for subsequent analyses (Supplementary Table S5). Further analysis was performed with 579 samples. PCA results were then plotted in R (v3.6.3). The ancestry analysis was run with ADMIXTURE (v.1.3.0) [17] by varying the number of ancestral populations K from 2 to 14. The best K was identified using the cross-error estimation implemented in ADMIXTURE. The results were plotted by using PONG (v1.4.7) [18] and AncestryPainter [19]. 

### 2.4. Ne and Divergence Time Analysis

The long-term *Ne* for the populations under study was estimated using R Package NeON [20] based on linkage disequilibrium patterns between SNPs. NeON starts with binary PLINK files and updates the genetic map information of the markers to calculate the *Ne* over time. It utilizes a relationship between the *Ne* and the average squared correlation coefficient of LD ($r_{LD}^{2}$) within predefined recombination distance categories between markers. We used a function that generates 250 overlapping recombination distance categories with a step of 0.001 centiMorgan (cM) from 0.005 to 0.25. The obtained *Ne* with a confidence interval 95% for each recombination distance category reflects *Ne* at a specific moment in the past. The long-term *Ne* was estimated as the harmonic mean of the effective population size along the generations in the past for each population [21]. Knowing the values of *Ne* and having the matrix of the calculated pairwise *F_ST_* values with 4P software [22], we could estimate the time of divergence between populations using the *diverg* function of the NeON R package. Divergence time in generations between pairs of study populations was estimated as follows:

T = ln(1 − *F_ST_*)/ln(1 − 1/2*Ne*),

where T represents divergence time. A generation is assumed to be 25 years long. The evolutionary history based on estimated divergence times was inferred using the UPGMA method implemented in MEGA X software ((v 10.2.5) [23]. 

### 2.5. Selection Signatures

Recent positive selection in the Lithuanian population was estimated using the cross-population extended haplotype-based homozygosity (XP-EHH) test [24] and the locus fixation index (*F_ST_*) [25], which was computed between Lithuanians and reference populations (Estonia, Belarus, Khanty, Mansi, Poland, Saami Kola, and Slovakia) at a given SNP. Genotyping data were phased for the analyses with SHAPEIT2 [26]. XP-EHH was run using selscan v1.2.0a [27]. *F_ST_* values were calculated with vcftools v.0.1.13 [28]. We kept SNPs with XP-EHH >2 as indicative of selection. Significant regions as candidates for positive selection were identified selecting any genomic region with two or more SNPs located in the top 0.1% of the XP-EHH empirical distribution and with at least one SNP presenting *F_ST_* *P*-value < 0.01. The regions under selection were annotated with ANNOVAR [29] using GRCh37 (hg19), dbSNP147 [30], RefSeqGene, and CADD (Combined Annotation dependent Depletin) version 1.347 [31]. The enrichment of biological processes in selected genes was tested using DAVID (Database for Annotation, Visualization, and Integrated Discovery) [32].

## 3. Results

### 3.1. Population Structure and Divergence Time Analysis

**Figure 1:**
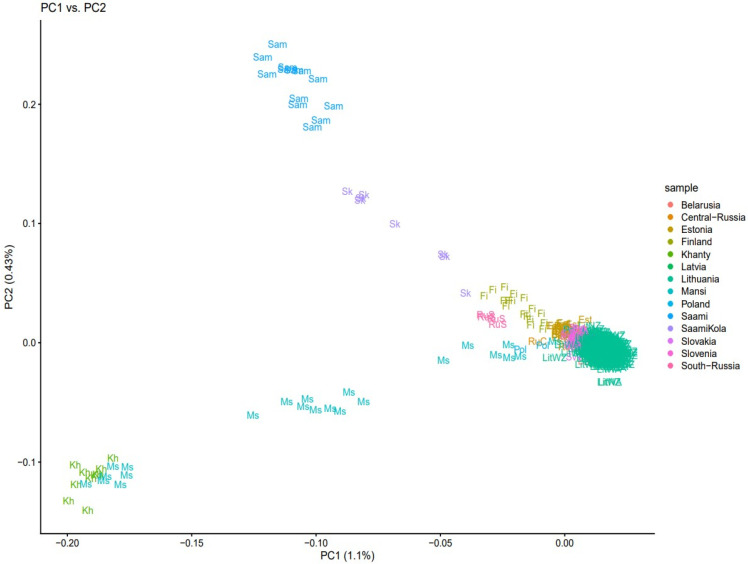
Principal component analysis of 105,853 SNPs in 579 individuals. The first principal component separates North-Eastern European, except the Finns, and Slavic populations from the rest.

To investigate the genetic diversity of the contemporary Lithuanian population among North-Eastern European (Latvia, Estonia, Belarus, Central Russia, South Russia, Finland, Poland, Saami, Saami Kola, Slovakia, and Slovenia) and West Siberian (Khanty, Mansi) populations, we first performed the principal component analysis (PCA) of 105,853 SNPs in 579 individuals (Figure 1). The first principal component separates North-Eastern European, except the Finns, and Slavic populations from the rest. Lithuanians clustered together with Latvians closer with Belarusians and Estonians in an intermediate position. Saami from Sweden and Saami from the Kola Peninsula grouped according to their demographic history with the Finns. Interestingly, Mansi is distributed in a wider range and some samples had positions in close proximity with the Baltic and Slavic populations (Figure 1). 

**Figure 2:**
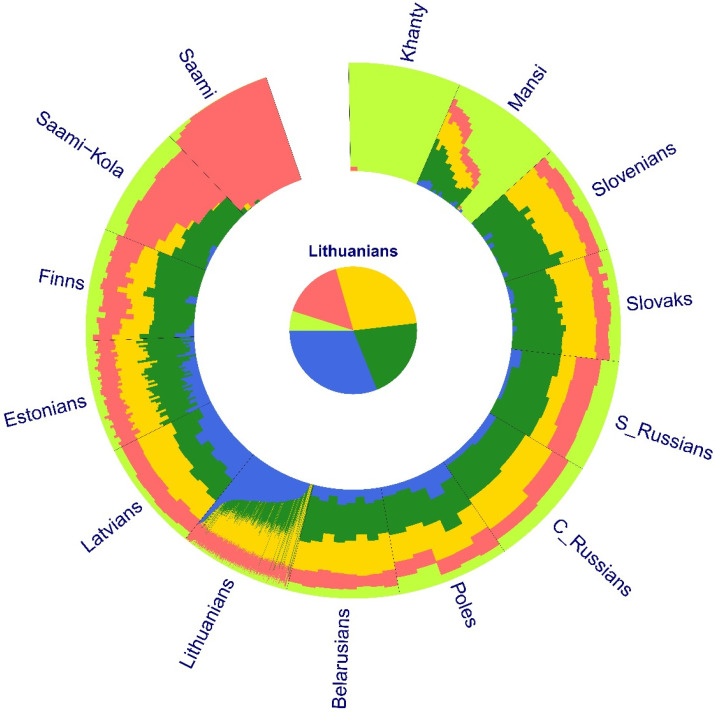
Population structure inferred from ADMIXTURE analysis on autosomal SNPs of Lithuanians and 13 external populations. The lowest cross-validation error (K = 5) shows the predominant genetic component (in blue) of Lithuanians.

The structure analysis was also supported by the ADMIXTURE plot (the best cross-validation error achieved at K = 5) (Figure 2, Supplementary Figure S1). The Lithuanians were characterized by predominant genetic component (red, 31% of the genetic ancestry) shared in larger proportion with Latvians (23.4%) and less with Estonians (12%), Belarusians (10%), and Poles (13.9%). This component, in a very small proportion (from 1% to 6%), can be seen in the rest of the analyzed populations and absent in Saami and Khanty. Many individuals from North-Eastern and Slavic populations share a proportion of their ancestry (green, blue, and purple) except for the Saami and Khanty isolates (Figure 2).

**Figure 3:**
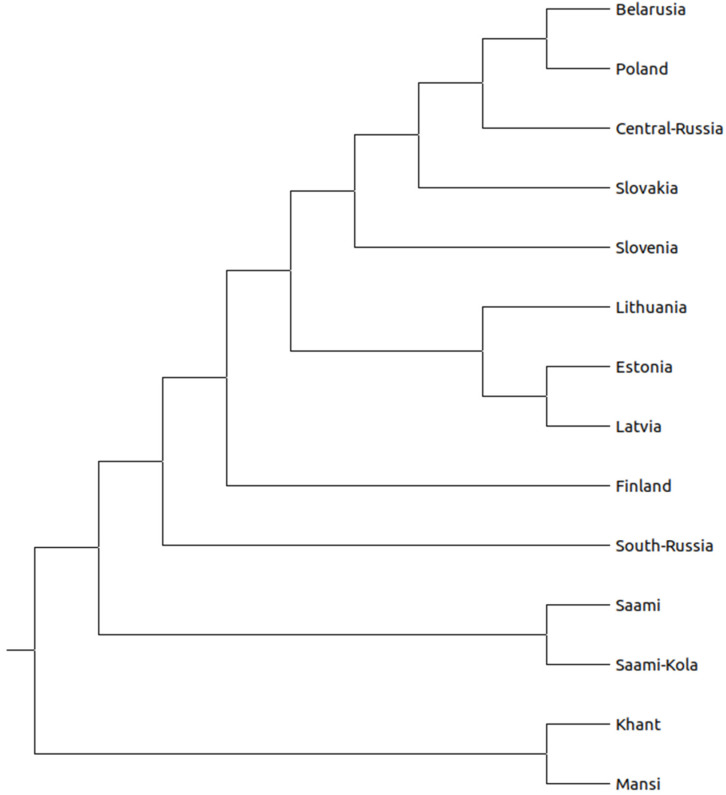
The evolutionary relationship based on the divergence time between populations was inferred using the UPGMA method. The analysis was conducted in MEGA X [19].

The divergence time between the studied populations was reconstructed by using the estimated *Ne* from LD patterns in genome-wide SNP using NeON [20] and the matrix of the calculated pairwise *F_ST_* values (Supplementary Tables S1 and S2), and visualized in the unweighted pair group method (UPGMA) phylogenetic tree constructed in MEGA X software (Figure 3). 

**Figure 4:**
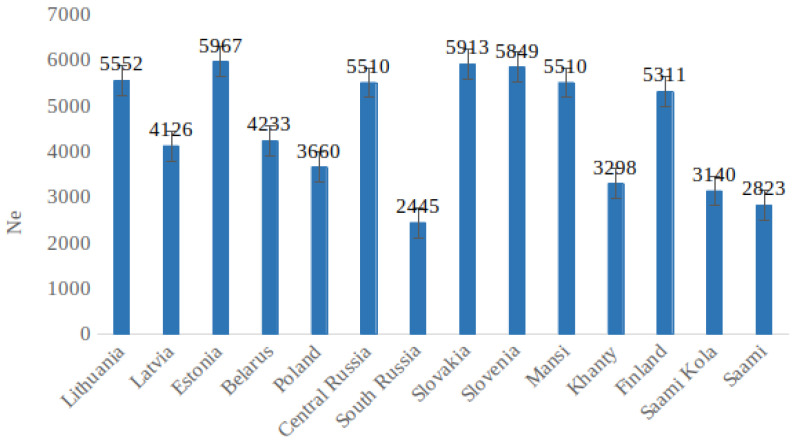
The harmonic mean of estimated *Ne* for each population. Error bars indicate 95% confidence intervals on each estimate.

The estimated *Ne* values ranged from 6000 to 200 generations ago, considering a generation time of 25 years (Supplementary Figure S2). The long-term *Ne* values, calculated as the harmonic mean [21], are summarized in Figure 4. We observed a variation in values from ~5900 in the Estonian population to ~2400 in the South Russian population. The UPGMA dendrogram shows the LD estimated time in generations of divergence between populations (Figure 3). The split times between the Lithuanians and other populations ranged from 240 to 12,871 YBP. The divergence between Lithuanians, West Siberians, and Saami was estimated to be ~9090 YBP) and ~6500 YBP, respectively. The split from Eastern European populations appears to have occurred around 1000 YBP, and from North-East Europe ~700 YBP (Supplementary Tables S3 and S4). The oldest split was observed between Khanty and Baltic populations including the Belarusian, Central Russian, Slovakian, and Slovenian populations in 12,250 YBP. Furthermore, the Mansi and Khanty groups formed an early diverging subclade, continued by the Saami group. As expected, all three Baltic populations formed a separate lineage with affinity to Slavs.

### 3.2. Identifying Regions under Recent Positive Selection

The identification of recent selective events was performed calculating XP-EHH and *F_ST_* between Lithuanians (LT) and Latvians (LVL), Estonians (EST), Mansi, Belarusians (BEL), Slovakians (SVK), Poles (POL), and Saami from the Kola Peninsula populations. Genome-wide distribution of detected signals between pairs of populations is shown in Supplementary Figure S3. We considered candidate regions with two or more SNPs present in the top 0.1% of the general distribution (based on XP-EHH results) and with at least one SNP with *F_ST_* *P* value < 0.01. Detected signals comprise a total of 24 candidate regions, with signatures of recent selection in the Lithuanian population (Table 1). 

Most recent positive selection signals were detected when comparing Lithuanians to the Latvians (five regions), Mansi (five regions), and Slovakians (five regions). One of the strongest signals identified in the LT–Mansi comparison was found in a 419 kb region in chromosome 1, which comprises the *SMYD3* gene coding a histone methyltransferase that functions in RNA polymerase II complexes by an interaction with a specific RNA helicase. The same region was detected when comparing LT–Khanty populations. Another signal detected in the LT–Mansi and LT–Khanty comparison was on chromosome 9, encompassing the *TYRP1* gene, which plays an important role in the melanin biosynthetic processes, however, no obvious functional SNPs were detected. When comparing LT–LVL populations, one strong signal was found in a 173 kb region in chromosome 11, which comprises the *MS4A* gene cluster encoding transmembrane proteins that are expressed in microglia. Two non-synonymous variants, rs10750931:154A > G and rs6591561:532A > G, in the *MS4A4A* gene were identified. The derived G allele at rs10750931 was present in low frequencies from 0.26 in Slovakians to 0 at Saami Kola, which was found in higher frequency in Latvians (0.43). The derived G allele at rs6591561 had low frequencies and was found at intermediate frequencies in Latvians and Poles. Interestingly, the rs6591561 variant was associated with reduced CSF sTREM2 (soluble triggering receptor expressed on myeloid cells 2) [33], elevated Alzheimer’s disease (AD) risk [34], and accelerated age-at-onset of AD [35]. In addition, comparing LT–LVL, a strong region was detected by XP-EHH analysis in chromosome 17, which contains the *NOS2* gene, produces nitric oxide (NO), which is a messenger molecule with diverse functions throughout the body. This gene carries a non-synonymous variant (rs2297518:1823C > T), which is associated with physical performance and longevity [36]. Among the recent positive selection signals present in the LT–SVK comparison, the 612 kb region containing the ZNF gene cluster (*ZKSCAN8*; *ZNF192P1*; *TOB2P1*; *ZSCAN9*; *ZKSCAN4*; *NKAPL*; *PGBD1*; *ZSCAN31*; *ZSCAN12*; *ZSCAN23*; *GPX6*; *GPX5*; *ZBED9*) located on chromosome 6 was selected. One non-synonymous SNP (rs12000:287A > G) in the *NKAPL* gene was identified. The same region encompasses olfactory receptor family genes. Regarding the LT–Saami from Kola Peninsula comparison, one of the strongest signals identified in the Lithuanian population was found at a 70 kb region in chromosome 14, which includes immune regulator genes *TCL1B* and *TCL1A*. A non-synonymous variant in *TCL1B* (rs1064017:277G > A) was identified among the top XP-EHH and *F_ST_* outliers along the region. This variant could contribute to the extreme IgE phenotype [37].

David 6.8 was used for enrichment analysis. We identified only one significantly enriched category in the BP ontology: GO:0035435, phosphate ion transmembrane transport, counting for two genes *SLC34A2*, *SLC37A1* (*P*-value 0.05). In the MF ontology, only one enriched category also satisfied the cut-off criteria, associated with sequence-specific DNA binding with six genes (*PGBD1*, *ZSCAN31*, *ZKSCAN8*, *ZSCAN9*, *ZSCAN12*, *ZSCAN23*), *p*-value 0.02. The enriched KEGG pathway was involved in thyroid hormone synthesis accounting with three genes (*PLCB4*, *GPX6*, *GPX5*), and *P*-value 0.01.

## 4. Discussion

There are still several unanswered questions about ancient events in the Lithuanian population. In the present research, we analyzed autosomal genome-wide SNPs to elucidate the genetic relationships between the Lithuanina and North-Eastern European and West Siberian populations. Through structure analysis, we both confirmed previously described findings and showed new findings between the Lithuanian and study populations. We identified the close genetic proximity of Lithuanians to neighboring populations (Latvians, Estonians, Belarusians) and in part with West Slavs (Poles, Slovaks, and Slovenians), however, with particular genetic distinctiveness. The Lithuanians overlap with these populations only in one extreme. South Russians were differentiated from the rest of the samples, positioning in the vicinity of Finns and Saami from the Kola Peninsula according to their demographic history. The PCA plot revealed genetic differentiation between Siberian and Saami groups, as those populations harbor specific genetic variation. The observed MANSI distribution in structure analysis and close proximity with the Baltic populations including Slovaks and Slovenians could be explained by detected gene flows between population ancestral to Mansi and North-Eastern European hunter–gatherers that occurred before 6.6–8000 years ago [38]. Genetic distance results showed moderate genetic drift between Khanty and North-Eastern and Eastern European populations (*F_ST_* ≥ 0.05). 

The admixture analysis also showed genetic distinctiveness of Lithuanians as a predominant genetic component (in blue) was found (Figure 2). This component was divided across the Lithuanian individuals and in a larger proportion found in the ethnolinguistic region of Zemaitija in Lithuania. Meanwhile, in the Lithuanian region Aukstaitija, another predominant genetic component (in yellow) and in lower proportion (in green) was found (Supplementary Figure S5). This latter component is also largely preserved in Slavic populations. The predominant genetic component of the Zemaitija region probably reflects the first early inhabitant ancestry of the Baltic region preserved in Lithuanians. According to Urnikyte et al. (2019), Lithuanians carry one of the highest proportion of the Western hunter–gatherer ancestry component compared to European populations [1]. Our findings are in agreement with Kushniarevich et al. (2015) [13], where Slavs share some genetic components with their neighboring Baltic populations. However, our genome wide SNP data results support a moderate impact of Finno-Ugrian populations on Lithuanians and Latvians.

We also estimated the long-term *Ne* in the North-Eastern European, Slavic, and West Siberian populations. The detected *Ne* of the North-Eastern European populations ranged from 4126 in Latvians to 5967 in Estonians, as expected because the *Ne* of many Northern populations is about 5000, indicating the bottlenecks associated with the last glacial period in Europe. The smallest *Ne* was identified in Saami (2323), South Russia (2445), Saami from the Kola Peninsula (3140), Khanty (3298), and Poland (3660). In Slavic populations, *Ne* was similar to North-Eastern European estimates. The demographic history of each population is shown in Supplementary Figure S4. 

The evolutionary relationships among populations visualized in the UPGMA tree from the divergence time matrix showed that separations occurred more recently for populations from the same geographical region (between Balts and Slavs). Longer divergence times separates West Siberian populations from non-Siberian populations. Moreover, phylogenetic tree shows more recent separations between East, West, and South Slavs except for the Poles. To the best of our knowledge, the separation patterns described were not reported in previous works using similar methods. 

We found evidence of selection in 24 regions, 21 of which had not been discovered in previous analyses of selection [1]. The strongest signatures of recent positive selection comprise the *TYRP1* gene involved in light skin pigmentation in Europeans [39] detected in Urnikyte et al. (2019) by comparing LT–CEU populations [1]. Recent selection signals can be related to the prevalence of some common diseases. Such signals point to the *MS4A4A* gene and non-synonymous variant rs659156, which are associated with reduced CSF sTREM2 (soluble triggering receptor expressed on myeloid cells 2) [33], increased Alzheimer’s disease (AD) risk [34], and accelerated age-at-onset AD [35]. The same signal was detected in previous scans for selection in Urnikyte et al. (2019) [1], indicating that occurrence of this gene in the Lithuanian population is not random. An interesting region with possible signature of selection was detected in chromosome 17. The non-synonymous variant (rs2297518:1823C > T) was identified inside the exon of the *NOS2* gene. Some studies found that this variant is associated with physical performance, longevity, increases risk of primary headaches [36,40], and can affect the susceptibility to arterial hypertension [13]. However, further studies are needed to predict functional relevance. We also detected several candidate genes related to immune response (*MS4A1*) and olfactory receptors (*OR1F12*, *OR2B6*), affecting odor perception in humans. The enrichment analysis with the selection signatures detected genes involved in phosphate ion transmembrane transport. One of those genes (*SLC37A1*) is a Pi-linked glucose-6-phosphate antiporter and appears to be involved in breast [41] and colorectal [42,43] cancers. A potential limitation of our study is the unequal and for some populations small sample sizes used, although with a small sample size, it is possible to recover the genetic diversity between populations. A larger sample size would provide more accurate results.

## 5. Conclusions

By analysing the genetic relationships between the Lithuania and North-eastern European and West Siberian populations we have identified the close genetic proximity of Lithuanians to neighbouring populations (Latvians, Estonians, Belarusians) and in part with West Slavs (Poles, Slovaks and Slovenians), however with particular genetic distinctiveness. Genome wide SNP data results support moderate impact of Finno-Ugrian populations on Lithuanians and Latvians. We have also estimated the long-term *Ne* in the North-eastern European, Slavic and West Siberian populations ranging from ~5900 in Estonian population to ~2400 in the South Russia population. The estimated divergence times between the Lithuanian and study populations ranged from 240 to 12,871 YBP. Furthermore we found evidence of selection in 24 regions, 21 of which have not been discovered in previous analysis of selection. 

Our study contributes to the progress of scientific knowledge in understanding the genetic diversity of geographically specific region and provides new insights into microevolutionary processes affecting local human populations.

## Figures and Tables

**Table 1:** Candidate regions for recent positive selection identified using XP-EHH and *F_ST_*.

Position	Genes	Population (SNPs *)
chr1:80069451-80662898	*ADGRL4*, *LINC01781*	LT-LVL(2)
chr1:245924864-246512218	*SMYD3*	LT-Khanty(3) LT-Mansi(12)
chr2:159061258-159558658	*CCDC148-AS1*, *CCDC148*, *PKP4*, *PKP4-AS1*	LT-LVL(7)
chr2:153248404-154751502	*FMNL2*, *RPRM*, *GALNT13*	LT-SVK(4)
chr4:25467149-25705912	*ANAPC4*, *LOC101929161*, *LOC101929161*, *SLC34A2*, *SEL1L3*	LT-EST(2)
chr6:24507761-24575094	*ALDH5A1*, *KIAA0319*	LT-EST(2)
chr6:625268-711792	*EXOC2*, *LOC101927691*	LT-LVL(3)
chr6:109742015-110156179	*PPIL6*, *AK9*, *FIG4*	LT-Mansi(4)
chr6:28018944-28630691	*OR2B6*, *OR1F12*, *ZKSCAN8*, *ZNF192P1*, *TOB2P1*, *ZSCAN9*, *ZKSCAN4*, *NKAPL*, *PGBD1*, *ZSCAN31*, *ZSCAN12*, *ZSCAN23*, *GPX6*, *GPX5*, *ZBED9*	LT-SVK(6)
chr7:19566286-20049554	*FERD3L*, *TWISTNB*, *TMEM196*, *LOC101927668*	LT-LVL(2)
chr8:60549318-61722552	*LOC100505501*, *CA8*, *CHD7*	LT-SaamiKola(8)
chr9:126324050-126690157	*DENND1A*	LT-POL(4)
chr9:12483221-12709305	*PTPRD-AS2*, *TYRP1*, *LURAP1L-AS1*	LT-Mansi(2)/ LT-Khanty(6)
chr11:60050125-60223018	*MS4A4A*, *MS4A6E*, *MS4A7*, *MS4A14*, *MS4A1*	LT-LVL(4)
chr11:83986071-86064757	*DLG2*, *PICALM*, *EED*, *HIKESHI*, *CCDC81*	LT-SVK(2)
chr12:19199330-19698168	*CAPZA3*, *PLEKHA5*, *AEBP2*, *LINC02398*	LT-Mansi(4)
chr12:27569063-28236948	*ARNTL2-AS1*, *SMCO2*, *PPFIBP1*, *KLHL42*, *PTHLH*, *LOC729291*	LT-SVK(2)
chr12:83895440-84204074	*TMTC2*, *SLC6A15*	LT-SVK(9)
chr14:96157187-96227199	*TCL1B*, *TCL1A*, *LOC107984703*, *TUNAR*	LT-SaamiKola(2)
chr16:82718030-82822631	*CDH13*, *LOC101928446*	LT-POL(2)
chr20:9325269-9380556	*PLCB4*	LT-BEL(2)
chr20:42488256-42593220	*GTSF1L*, *LINC01728*, *TOX2*	LT-Mansi(4)
chr21:21388493-21644798	*LINC01683*, *LINC02573*	LT-SaamiKola(6)
chr21:43992477-44066201	*SLC37A1*, *LINC01671*, *PDE9A*	LT-SVK(3)

* Number of significant SNPs located at the top 0.1% of the empirical distribution for the XP-EHH and at least one SNP in the region had *F_ST_* *P* value < 0.01.

## Data Availability

The Lithuanian dataset is available in the Figshare repository: https://figshare.com/articles/dataset/Patterns_of_genetic_structure_and_adaptive_positive_selection_in_the_Lithuanian_population_from_high-density_SNP_data/7964159.

## Supplementary Materials

The following are available online at https://www.mdpi.com/article/10.3390/genes12111730/s1, Figure S1. Map of Lithuanian ethnolinguistic groups. Six regions based on dialect are distinguished in Lithuania: three groups from Aukstaitija (West, South, and East) and three groups from Zemaitija (North, West, and South). Figure S2. Admixture analysis of Lithuanians and 13 external populations. Admixture results from K = 2 to K = 7. The lowest cross-validation error in the analysis was K = 5. Individuals are represented as vertical colored bars, in which each different colored segment represents the proportion of an individual’s ancestry derived from one of the K populations. Figure S3. Plots of variation in Ne estimates for each population. The x-axes show the time measured in generations; the y-axes show *Ne* values with the confidence intervals (5th and 95th percentile). Figure S4. Manhattan plots of -log10 transformed XP-EHH p-values across the autosomes. Figure S5. Admixture analysis of Lithuanians and 13 external populations was performed by AncestryPainter [1] with five ancestral source populations. (A) The ethnolinguistic region of Lithuania, North Zemaitija (NZ), is highlighted at the center of the graph. (B) The ethnolinguistic region of Lithuania, South Aukstaitija (SA), is highlighted in the center of the graph. EA—East Aukstaitija, SA—South Aukstaitija, WA—West Aukstaitija, NZ—North Zemaitija, SZ—South Zemaitija (SZ), WZ—West Zemaitija. Table S1. Estimated long-term *Ne* values for the studied populations. Table S2. Weir and Cockerham *F_ST_* estimates between pairs of studied populations. Table S3. Calculated divergence time in years between pair of continents. Table S4. Calculated divergence time in years with 95% CI between pair of continents. Table S5. Lithuanian individuals excluded from the selection analyses.
