# Supplementary material for: Genome-Wide Landscape of North-Eastern European Populations: A View from Lithuania
Source: Genes (Basel). 2021 Oct 28;12(11):1730. doi: 10.3390/genes12111730 (PMC8623362; doi:10.3390/genes12111730)
Supplement: Supplementary file 1 [file genes-12-01730-s001.zip › supplemenary/Suppl Figures_Urnikyte.pdf]

Supplementary Material

Genome-wide landscape of north-eastern European populations: a view from Lithuania

Alina Urnikyte[1,*], Alma Molyte[1,2] and Vaidutis Kučinskas[1]

[1]Department of Human and Medical Genetics, Institute of Biomedical Sciences, Faculty of Medicine, Vilnius University, Santariškiu St. 2, LT-08661 Vilnius, Lithuania; alma.molyte@mf.vu.lt (A.M.); vaidutis.kucinskas@mf.vu.lt (V.K.)

[2]Department of Information Systems, Faculty of Fundamentals Sciences, Vilnius Gediminas Technical University Saulėtekio al. 11, LT-10223 Vilnius, Lithuania

*Correspondence: alina.urnikyte@mf.vu.lt (A.U.); Tel.:+37069855292

Supplementary Figures

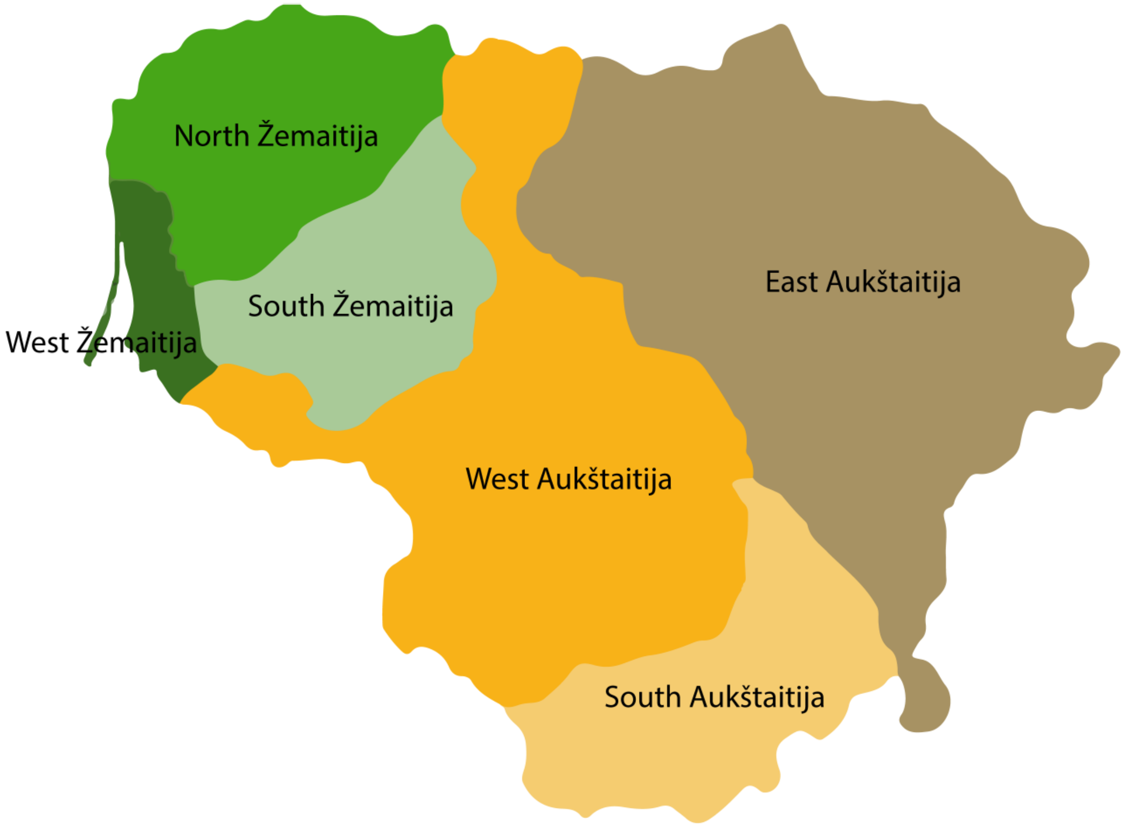

Figure S1. Map of Lithuanian ethnolinguistic groups. Six regions based on dialect are distinguished in Lithuania: three groups from Aukstaitija (west, south and east) and three groups from Zemaitija (north, west and south).

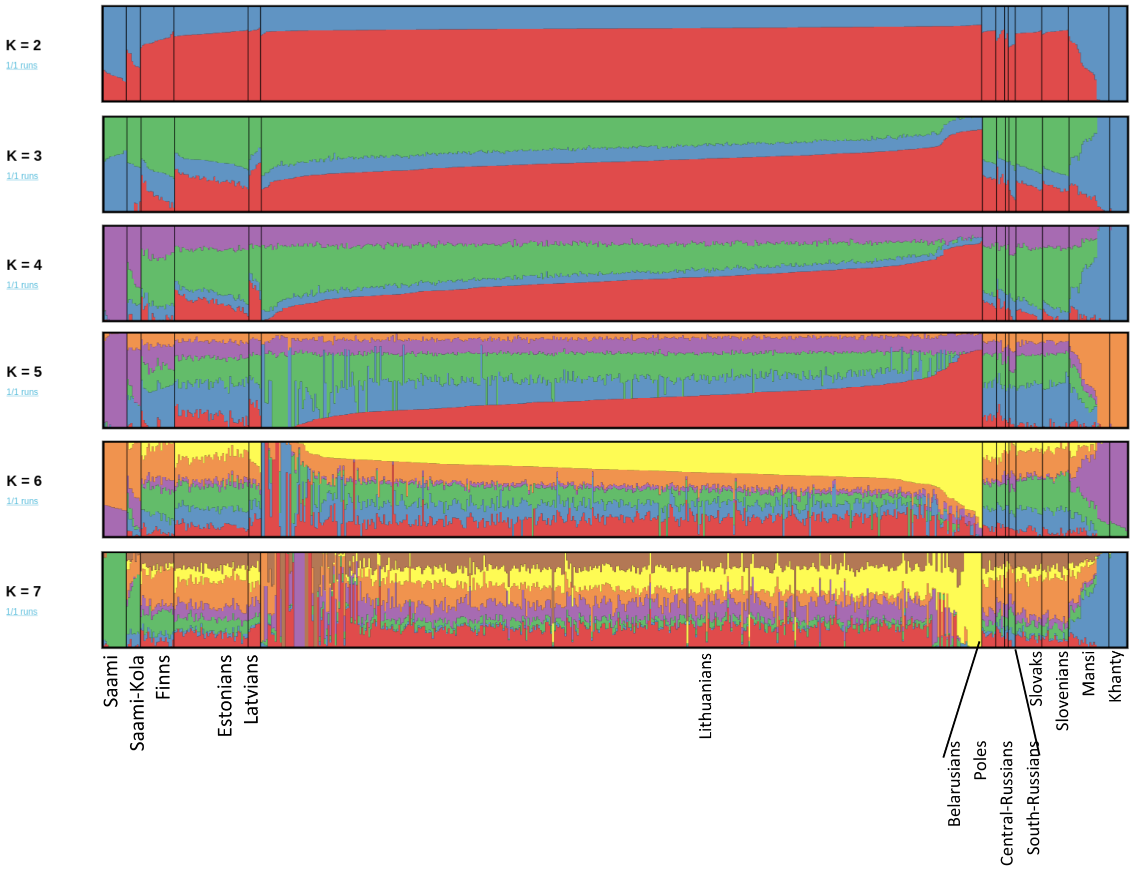

Figure S2 Admixture analysis of Lithuanians and 13 external populations. Admixture results from K=2 to K=7. The lowest cross-validation error in the analysis was K = 5. Individuals are represented as vertical coloured bars, in which each different coloured segment represents the proportion of an individual's ancestry derived from one of the K populations.

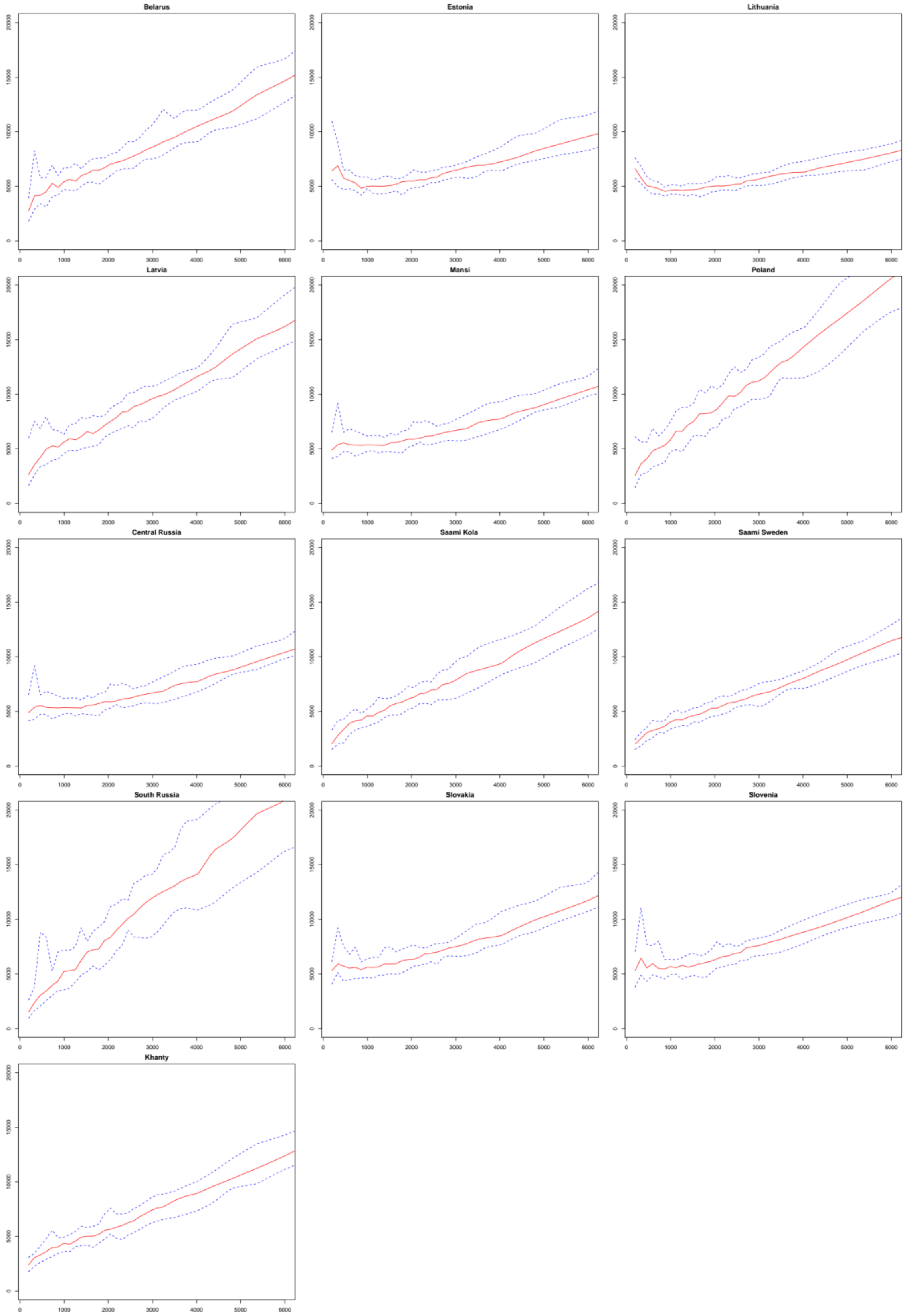

Figure S3. Plots of variation in *Ne* estimates for each population. The x-axes show the time measured in generations; the y-axes show *Ne* values with the confidence intervals (5th and 95th percentile).

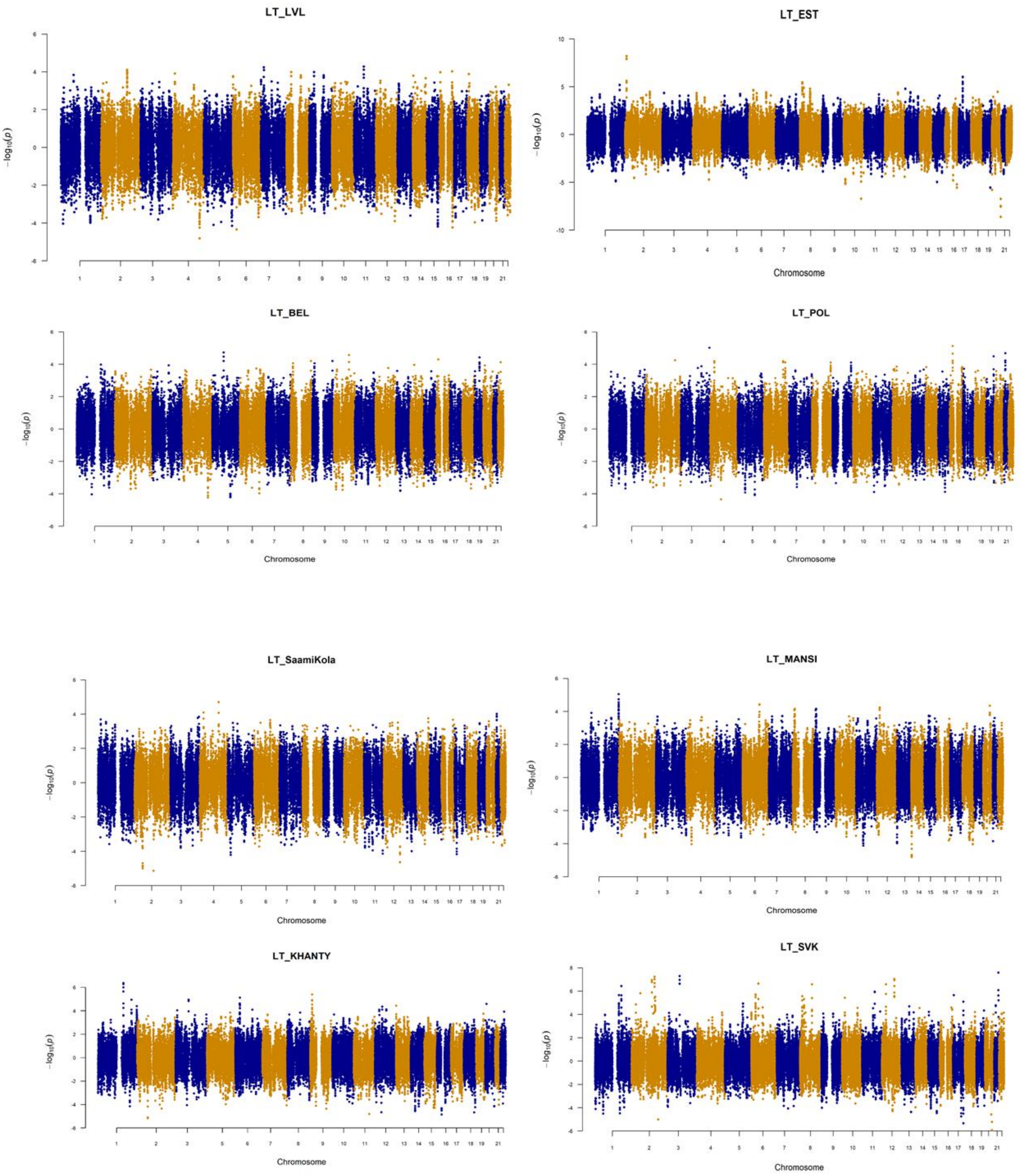

Figure S4. Manhattan plots of -log10 transformed XP-EHH p-values across the autosomes.

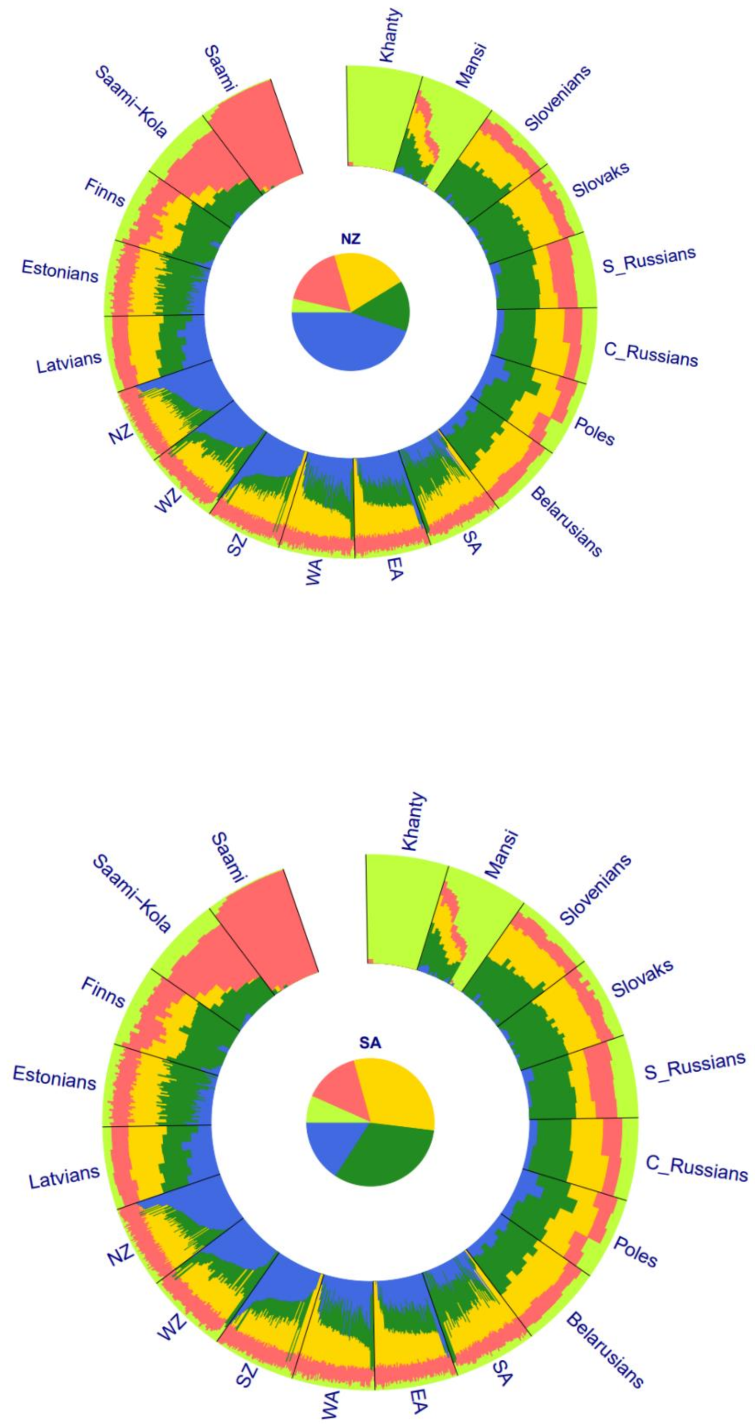

Figure S5. **Admixture analysis of Lithuanians and 13 external populations performed by Ancestry Painter** [1] with 5 ancestral source populations. A) The ethnolinguistic region of Lithuania, North Zemaitija (NZ), is highlighted in the centre of the graph. B) The ethnolinguistic region of Lithuania, South Aukstaitija (SA), is highlighted in the centre of the graph. EA – East Aukstaitija, SA - South Aukstaitija,

WA – West Aukstaitija, NZ – North Zemaitija, SZ – South Zemaitija (SZ), WZ –West Zemaitija.

References

1. Alexander DH, Novembre J, Lange K. Fast model-based estimation of ancestry in unrelated individuals. Genome Res. 2009;19:1655–64.
